# Linking genes with ecological strategies in *Arabidopsis thaliana*

**DOI:** 10.1093/jxb/ery447

**Published:** 2018-12-15

**Authors:** Margarita Takou, Benedict Wieters, Stanislav Kopriva, George Coupland, Anja Linstädter, Juliette De Meaux

**Affiliations:** 1 Institute of Botany, University of Cologne, Germany; 2 Max Planck Institute of Plant Breeding Research, Cologne, Germany; 3 Institute of Crop Science and Resource Conservation (INRES), University of Bonn, Germany

**Keywords:** *Arabidopsis thaliana*, CSR strategy, local adaptation, natural variation, trait syndrome

## Abstract

*Arabidopsis thaliana* is the most prominent model system in plant molecular biology and genetics. Although its ecology was initially neglected, collections of various genotypes revealed a complex population structure, with high levels of genetic diversity and substantial levels of phenotypic variation. This helped identify the genes and gene pathways mediating phenotypic change. Population genetics studies further demonstrated that this variation generally contributes to local adaptation. Here, we review evidence showing that traits affecting plant life history, growth rate, and stress reactions are not only locally adapted, they also often co-vary. Co-variation between these traits indicates that they evolve as trait syndromes, and reveals the ecological diversification that took place within *A. thaliana*. We argue that examining traits and the gene that control them within the context of global summary schemes that describe major ecological strategies will contribute to resolve important questions in both molecular biology and ecology.

## Teaser

This review collects together evidence that phenotypic traits often evolve as syndromes in *Arabidopsis thaliana* populations, pointing to extensive levels of ecological diversification within species.

## Local adaptation suggests a diversity of ecological specializations within *A. thaliana*

*Arabidopsis thaliana* (L.) Heyhn is exceptional within its genus. It is the only annual species, it has adapted to open, dry habitats prone to seasonal drought (Ruppert *et al.*, 2015; Kiefer *et al.*, 2017), and its reproductive success is directly dependent on interannual variation in environmental conditions (Segrestin *et al.*, 2018). It also has the widest geographic range in the Arabidopsis genus (Clauss and Koch, 2006; Novikova *et al.*, 2016). Natural populations have been found throughout Europe, from the North of Scandinavia to the South of Spain, in the Balkans, in Central Asia, China, and parts of Africa (Hoffmann, 2005; He *et al.*, 2007; 1001 Genomes Consortium. 2016; Durvasula *et al.*, 2017). It is also naturalized in North America and Argentina (Stock *et al.*, 2015; Kasulin *et al.*, 2017; Exposito-Alonso *et al.*, 2018*a*). This exceptionally wide distribution range is only limited by very low spring or autumn temperatures or by high temperature in regions of low precipitation (Hoffmann, 2002).

The unrivaled genomic resources available for these populations have helped demonstrate that the last glacial period determined the current distribution of genetic variation (reviewed in Koch, 2019). After the last glacial maximum, populations have spread towards Northern latitudes, experiencing successive bottlenecks (Durvasula *et al.*, 2017; Svardal *et al.*, 2017). As a result, regional diversity is highest in Africa and lowest in Scandinavia. Genetic variation in Eurasia also follows a longitudinal gradient (1001 Genomes Consortium, 2016; Zou *et al.*, 2017).

The local adaptation of *A. thaliana* populations has been documented throughout its range, despite a history of pervasive gene flow (Fournier-Level *et al.*, 2011; Hancock *et al.*, 2011; Agren and Schemske, 2012; Savolainen *et al.*, 2013; Weigel and Nordborg, 2015; Svardal *et al.*, 2017). Field experiments and correlation analyses with climate parameters identified numerous genomic regions associated with local climatic conditions. Association studies correlating single nucleotide polymorphism (SNP) variants with climatic variation showed that non-synonymous SNPs were enriched among SNPs associating with environmental variance (Hancock *et al.*, 2011; Lasky *et al.*, 2014). Among them, SNPs associating with fitness differences in the field were also over-represented (Hancock *et al.*, 2011). Furthermore, alleles associating with fruit production are more frequent in populations closer to field sites where the selective advantage was expressed (Fournier-Level *et al.*, 2011). Therefore, it is now clear that much of the variation found in this species has played a role in optimizing plant performance to local environmental conditions.

## A combination of development traits underpins local adaptation in *A. thaliana*

Flowering time is one of the development traits underpinning adaptation in *A. thaliana*. It has been extensively studied, and elevated levels of variation have been observed in the lab (Koornneef *et al.*, 2004). The adaptive relevance of its genetic variation is supported by multiple independent studies. Variation in flowering time follows climatic clines, at both the regional and species levels (Mendez-Vigo *et al.*, 2011; Montesinos-Navarro *et al.*, 2011; Debieu *et al.*, 2013; Li *et al.*, 2014; Sasaki *et al.*, 2015). Warmer climates appear to favor earlier flowering time, a pattern that has been documented for a great number of species (Austen *et al.*, 2017; Whittaker and Dean, 2017). Strong selection for early flowering was detected in Italy but was weaker in Sweden (Ågren *et al.*, 2017). Population genetics studies also uncovered signatures of natural selection on genes controlling flowering time (Le Corre, 2005; Toomajian *et al.*, 2006). Finally, the analysis of co-variation between environmental and phenotypic variance consolidated evidence for the adaptive distribution of this trait (van Heerwaarden *et al.*, 2015).

Much of the flowering time variation measured in the lab, however, does not manifest as variation in flowering phenology in the field (Wilczek *et al.*, 2009; Brachi *et al.*, 2010; Hu *et al.*, 2017). It is indeed tightly dependent on the environmental conditions prevailing during seedling establishment, and hence on another developmental trait: the timing of germination (Donohue, 2002; Wilczek *et al.*, 2009). Both field experiments and theoretical models integrating seed and flowering phenology have shown that seed dormancy is often decisive for controlling the life cycle across environments (Chiang *et al.*, 2013; Burghardt *et al.*, 2015). Therefore, the adaptive relevance of variants modulating flowering time control must be examined in the context of variation for the timing of germination.

There is indeed consistent support for the adaptive relevance of traits determining the timing of germination. Seed dormancy has a strong fitness advantage before the hot season, but can impair fitness if it delays plant growth before winter (Donohue, 2002; Donohue *et al.*, 2005; Chiang *et al.*, 2013). Population genetics analysis of seed dormancy and its major quantitative trait locus (QTL) *DOG1* supported the adaptive importance of strong dormancy in Southern regions, to escape dry and hot summers, whereas weaker dormancy was reported in Norway, where the season is shorter (Kronholm *et al.*, 2012; Postma and Ågren, 2016; Kerdaffrec *et al.*, 2016).

Since flowering time determines the maternal environment the seeds experience during their maturation, it also impacts life history traits expressed by the next generation (Chiang *et al.*, 2013; He *et al.*, 2014; Postma and Ågren, 2015). Light, temperature, nutrient availability, and water status have all been identified as significant environmental factors influencing the maternal inheritance of seed dormancy (Footitt *et al.*, 2013; He *et al.*, 2014; Morrison and Linder, 2014; Kerdaffrec *et al.*, 2016). Germination can also be distributed over more than one seasonal window. For example, maintaining a spring germinating cohort is important for the maintenance of populations exposed to low winter temperature (Picó, 2012; Akiyama and Ågren, 2014). Furthermore, later flowering can lead to late seed dispersal, which can result in overwintering at the seed stage (Hu *et al.*, 2017).

Flowering time and seed dormancy are therefore jointly subject to fluctuating seasonal selective forces. They can evolve as a syndrome, defining distinct life history strategies that have diversified across environments (Chiang *et al.*, 2013; Vidigal *et al.*, 2016; Marcer *et al.*, 2018). An analysis of seed dormancy and flowering time co-variation revealed that the optimization of the two traits probably depends on latitudinal differences in climate. Late flowering (i.e. vernalization requirement) and strong dormancy are more frequent in regions where summer drought is typically more severe, whereas late flowering in Northern latitudes co-varies negatively with dormancy (Debieu *et al.*, 2013). Co-variance between flowering time and dormancy is also detected at a much smaller scale, along steep altitudinal gradients (Vidigal *et al.*, 2016). Normally, diverse life history trait combinations can allow comparable population growth rates in field conditions (Taylor *et al.*, 2017). In some years, however, early winter frost can wipe out genotypes expressing inadequate life histories (Hu *et al.* 2017). Minimum winter temperature and precipitation, in fact, were also the main climatic factors that acted as selective pressures on flowering time and their underpinning genes in a set of Iberian *A. thaliana* genotypes (Méndez-Vigo *et al.*, 2011). This suggests that extreme deviation from seasonal averages may be important drivers of the allelic combination of life history variants adjusting development to the optimal growth season throughout the species range.

## Patterns of co-variation between growth rate and developmental traits suggest the existence of trait syndromes

Beyond the combination of life history traits to target the best season for growth, *A. thaliana* also displays considerable genetic variation in its growth rate (Debieu *et al.*, 2013; Marchadier *et al.*, 2018, Preprint). The pattern of co-variation linking growth rate with flowering time and seed dormancy is independent of population structure, and changes from Southern to Northern latitude (Debieu *et al.*, 2013). This suggests that trade-offs between growth rate and life history change across the distribution range of the species (Debieu *et al.*, 2013). It further implies that complex multitrait combinations (i.e. trait syndromes) are necessary to adjust to the changing trade-offs imposed by regional differences in climatic conditions. Co-variation between flowering time, final biomass, and average rate of biomass accumulation before flowering also suggests that genetic adaptation to local climate conditions is mediated by a trait syndrome (Vasseur *et al.*, 2018*a*).

Genetic variation for tolerance to drought stress, just like that for life history, displays signatures of local adaptation. Many genetic variants have been identified that also affect either root or rosette growth in the face of severe drought stress (El-Soda *et al.*, 2015; Clauw *et al.*, 2016; Davila Olivas *et al.*, 2017). Several studies highlighted the adaptive relevance of variation in the ability to maintain growth and photosynthesis when water is limited. After accounting for the demographic history of the species, stomatal size variation was found to correlate with water-use efficiency (i.e. rate of carbon fixation to water loss) and both air humidity and the local probability of spring drought severity (Dittberner *et al.*, 2018), in agreement with field measurements (Mojica *et al.*, 2016). The molecular evolution of the gene P5CS, which contributes to the synthesis of proline, a potent osmoprotectant in *A. thaliana*, suggests that it contributed to local adaptation (Kesari *et al.*, 2012). Nucleotide variants within genes displaying stress-dependent expression were also shown to be over-represented among variants correlating with climatic parameters (Lasky *et al.*, 2014; Exposito-Alonso *et al.*, 2018*c*).

In fact, genetic variation for stress tolerance not only is involved in local adaptation, but it also appears to be part of a trait syndrome, because it is often reported to coincide with variation in life history. Early flowering individuals, which sometimes complete their life cycle within a few weeks, can escape conditions causing high pre-reproductive mortality (Franks, 2011; Fournier-Level *et al.*, 2013; Riboni *et al.*, 2013). In addition, the major flowering time QTL *FRIGIDA* controls not only the timing of flowering but also water-use efficiency (Johanson *et al.*, 2000; Lovell *et al.*, 2013). Improved performance in plants exposed to moderate drought stress is correlated with the ability to flower early (Bac-Molenaar *et al*., 2016), but genotypes with a strong vernalization requirement tend rather to avoid the effect of drought by maintaining their internal water level (McKay *et al.*, 2003; Des Marais *et al.*, 2012; Lovell *et al.*, 2013; Easlon *et al.*, 2014; Davila Olivas *et al.*, 2017). The most stress-tolerant individuals actually appear to be either early flowering or slow growing (Davila Olivas *et al.*, 2017; Vasseur *et al.*, 2018*a*).

Because of its co-variation with life history, adaptation to drought stress can show counter-intuitive patterns. In *A. thaliana*, local adaptation for increased tolerance to drought stress is not found in the driest regions, because, in these areas, natural selection promoted genotypes with the ability to escape the stress (Kronholm *et al.*, 2012; Vidigal *et al.*, 2016, Mojica *et al.*, 2016; Tabas-Madrid *et al.*, 2018). Genotypes showing smaller stomata, higher water-use efficiency, and longer photosynthetic activity in the face of terminal drought have in fact evolved in Southern Scandinavia, where the growth season is too short to allow an escape from the drier season but dry enough to require improved drought tolerance (Mojica *et al.*, 2016; Dittberner *et al.*, 2018; Exposito-Alonso *et al.*, 2018*c*). Genotypes with a strong vernalization requirement are in fact more frequent in this region, limiting the possibility to escape drought during the growing season (Li *et al.*, 2014). Non-monotonic patterns of co-variation between flowering time and temperature have also been reported in Spain (Tabas-Madrid *et al.*, 2018), suggesting that the selective advantage of early flowering for persisting in dry regions depends on a broader ecological context, and thus presumably on the possibility to rely on earlier flowering to escape stressful conditions. The evolution of the response to abiotic stress in *A. thaliana* is therefore not independent of the evolution of the timing of life history transitions.

The ability of plants to face biotic stresses is also dependent on life history variation. Alleles accelerating flowering were shown to be often combined with alleles decreasing the expression of plant defense genes throughout natural *A. thaliana* populations (Glander *et al.*, 2018). An indication that this assortment coincides with differential fitness suggests that it has been driven by natural selection. In addition, plant growth in response to the specialist herbivore *Pieris rapae* was enhanced in fast-flowering individuals but showed a trade-off with the drought response (Davila Olivas *et al.*, 2017). Here again, this hints at the evolution of a trait syndrome, where early flowering genotypes may have been selected for their ability to allocate fewer resources into defense, in order to maximize their growth rate or to reshuffle energetic priorities and ensure survival.

**Fig. 1.**
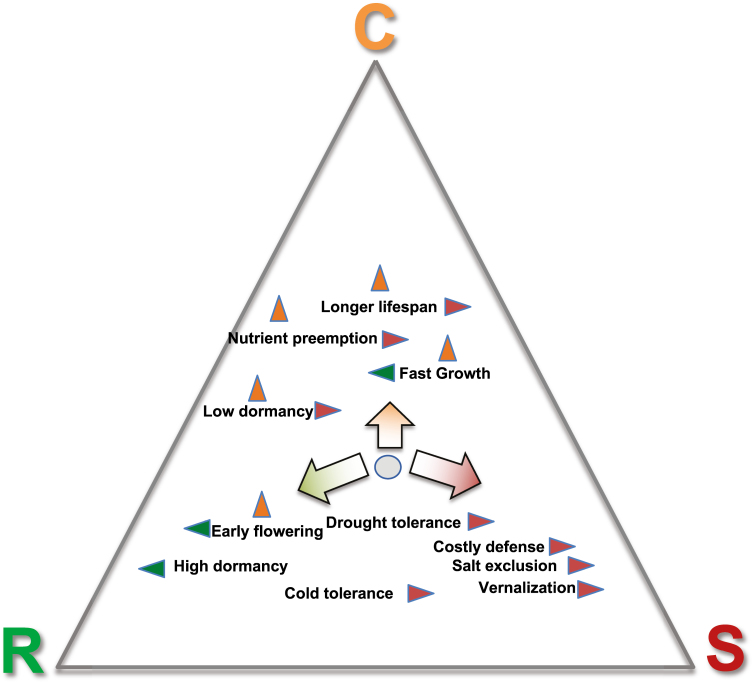
Hypothetical contribution of adaptive traits to ecological diversification within *Arabidopsis thaliana*. The diversity of ecological strategies can be summarized in Grime’s C–S–R strategy triangle which reflects a major trade-off between competitive, stress-tolerant, and ruderal strategies. Large arrows represent the major evolutionary trajectories. Intraspecific variation along the R–S axis has been documented in *A. thaliana*. Fewer studies document intraspecific variation along the R–S and C–S axes. Small arrows represent the contribution of individual traits (green, contribution to increased ruderality; red, contribution to increased stress tolerance; orange, contribution to increased competitive ability). The position of the trait along the vertical axis is dictated by graphical constraints only. The gray dot symbolizes a genotype with a given strategy. Large arrows point to possible ecological shifts that populations can evolve. These shifts can be operated by concomitant trait shifts.

*Arabidopsis thaliana* thus displays significant levels of genetic variation in traits controlling life history, growth rate, or tolerance to diverse stresses, all of which co-vary with each other and with climatic conditions at the location of origin. This suggests that adaptation to novel environments after the last glaciation has allowed the evolution of trait syndromes (i.e. a combination of multiple traits). These combinations probably reflect both adaptive synergies and global trade-offs imposed by resource limitations. Identifying the exact composition of trait syndromes and their variation requires a careful monitoring of life history transitions, growth rates, stress tolerance, and plant fitness in natural conditions. We argue below that interpreting trait variation and co-variation in the global context of plant ecological strategies, within summary schemes developed by ecologists to describe the major dimensions determining variation in form and function within and across habitats, may help resolve the ecological significance of traits and their combinations (Fig. 1).

## Interpreting *A. thaliana* trait syndromes in the context of major ecological strategies

Not all possible trait combinations are viable in natural environments: natural selection indeed limits the diversity of forms and functions in plants as a result of trade-offs among the diverse options for resource allocation (Reich, 2014; Díaz *et al.*, 2016). This major constraint has motivated several attempts to classify plants with respect to their ecological strategies (reviewed in Westoby *et al.*, 2002; Díaz *et al.*, 2016). Among them, Grime’s CSR theory (Grime, 1974, 1977) is a prominent strategy scheme (Pierce *et al.*, 2017). It distinguishes three primary strategies, namely competitive (C), stress-tolerant (S), and ruderal (R). The first two strategies evolve in rather constant environments, which differ in the severity of resource shortage (light, water, and/or nutrients). The third one prevails in disturbed environments, and involves investment of a large proportion of resources in propagules from which the population can regenerate in the face of repeated biomass destruction events. Distinct strategies may also co-occur within a given environment, enhancing local niche separation between species (Kraft *et al.*, 2008). The multivariate and complex traits that form the basis of ecological strategies are often difficult to measure. Yet, a small number of plant functional traits related to growth, survival, and reproduction have been shown to summarize efficiently the overall diversity of plant life form and functions (Díaz *et al.*, 2016). Among them, as many as three leaf traits—leaf area, leaf dry matter content, and specific leaf area—can be used as surrogate to describe a species’ CSR strategy (Pierce *et al.*, 2017).

Like many other annuals in the Brassicaceae family, these leaf traits position *A. thaliana* as a typical R-strategist (Pierce *et al.*, 2017). It is typically encountered in regularly disturbed habitat patches, such as urban, ruderal, or mountainous habitats, and its seedlings are directly exposed to seasonal climatic fluctuations (Picó *et al.*, 2008; Bomblies *et al.*, 2010; Svardal *et al.*, 2017). However, the past years have shown that plant species are far from having a fixed CSR strategy. On the contrary, strategy classifications at the species level have been more and more challenged by the large spectrum of intraspecific variation (Des Roches *et al.*, 2018; Volaire, 2018). For this reason, it is now increasingly acknowledged that trade-offs in life history and growth strategies can also occur at the level of genotypes and populations, and that more attention should be devoted to interindividual and interpopulation variation of the CSR strategy (Astuti *et al.*, 2018). Intraspecific trade-offs have been found along the S–R axis of Grime’s CSR strategy scheme (Bilton *et al.*, 2010; May *et al.*, 2017), but also along the C–S axis (Ravenscroft *et al.*, 2014; Astuti *et al.*, 2018). These trade-offs are commonly explained as a mechanism of local adaptation, for example in response to different levels of resource stress. It is thus not surprising that considerable intraspeciﬁc variation has also been found for *A. thaliana.* A study with 16 individual accessions sampled along a steep altitudinal gradient revealed that populations from hotter climates clustered towards the stress-tolerant end of the observed strategy spectrum, implying pronounced intraspecific variation along the S–R axis (May *et al.*, 2017). The extent of variation along the S–R axis was recently confirmed by a comprehensive analysis of variation in CSR positioning in some 300 genotypes in *A. thaliana* (Vasseur *et al.*, 2018*b*). As for other annual plants, we could thus assume that *A. thaliana* populations growing in water- or temperature-limited habitats may be well adapted to high levels of stress and thus characterized as stress tolerant (Volaire, 2018). In contrast, genotypes that grow fast and complete their life cycle within a few weeks may be described as extreme ruderals (Fig. 1). In *A. thaliana*, genotypic variation covers the whole S–R strategy spectrum. The geographical distribution of this variation contrasts with that of genome-wide patterns of variation, suggesting a role in local adaptation (Vasseur *et al.* 2018*b*).

Intraspecific variation along the S–C or R–C axes involves traits that have not been intensively investigated in *A. thaliana* (Fig. 1). The ability to compete with other species plays a presumably minor role for a pioneer species that only subsists in disturbed environments. Yet, the few studies that investigated this aspect (e.g. Masclaux *et al.*, 2010; Baron *et al.*, 2015; Frachon *et al.*, 2017) suggest that intraspecific variation in C-strategic features will also be significant (Fig. 1). The disturbed environments in which *A. thaliana* can be found are sometimes densely populated (G. Schmitz, personal communication; see also the population studied in Frachon *et al.*, 2017). Interspecific competition has been shown to modify the pattern of natural selection operating on flowering traits in a collection of recombinant inbred lines (Brachi *et al.*, 2012). Some *A. thaliana* genotypes, initially collected in a densely populated habitat patch, displayed the ability to decrease the biomass of some of their interspecific competitors (Baron *et al.*, 2015; Frachon *et al.*, 2017).

Intraspecific competition is also expected to stand under strong selection in the species. The population census size is small in a newly colonized habitat patch but will increase with the age of the habitat patch. Under increasing density of intraspecific competitors, plants differ in their ability to reach the fruiting phase and produce seeds (Masclaux *et al.*, 2010; Muñoz-Parra *et al.*, 2017). Root growth is also negatively impacted by intraspecific competition (Muñoz-Parra *et al.*, 2017). Competitive ability may also modulate the intensity of selection on water-use efficiency variants (Campitelli *et al.*, 2016). Intraspecific variation along the S–C or R–C axes might be less pronounced than along the S–R axis, but is probably not negligible, as indicated by the recent discovery of a gene locus promoting positive interactions between genotypes (Wuest and Niklaus, 2018, Preprint; see also Vasseur *et al.*, 2018*b*).

The CSR strategy scheme is one of the conceptual frameworks that can help understand the role that specific trait (or gene) combinations have played in the ecological diversification observed within *A. thaliana*. To date, their contribution remains mostly hypothetical (Fig. 1). Late flowering in controlled conditions was reported to associate with increased stress tolerance in the CSR scheme, yet whether this trait mediates an increase in stress tolerance or associates with traits which control stress tolerance has not been elucidated (Vasseur *et al.*, 2018*b*). Identifying causal links between traits, their underpinning genes, and shifts in CSR strategy could considerably ameliorate knowledge transfer between model and non-model species, because this scheme was designed to facilitate interspecific comparisons (Pierce *et al.*, 2017).

## Towards linking molecular biological functions with their role in ecological strategies

Exploring how traits are combined in natural populations to tune the ecological strategy of local genotypes to their local environmental conditions can indeed cast new light on gene function at the molecular level. We illustrate this idea with two points: first we describe how natural variation can help identify genes controlling ecologically important traits and focus on plant nutrition as an exemplary trait. Secondly, we show that the function of the well-known flowering time regulator FLOWERING LOCUS C (FLC) can be revised in the perspective of ecological strategies.

Studies of natural variation have greatly assisted the discovery of the genes controlling functions that cannot be easily dissected in mutant screen approaches (reviewed in Alonso-Blanco *et al.*, 2009). For example, QTL analyses of nutrients unraveled the function of the anion channel AtCLC-c in nitrate transport to vacuoles (Loudet *et al.*, 2003; Harada *et al.*, 2004) or showed that the ATPase subunit G and the multicopper oxidase LPR1 have a major impact on the accumulation of phosphate and phytate (Bentsink *et al.*, 2003; Reymond *et al.*, 2006). They further showed that foliar sulfate accumulation is dependent on sulfate reduction rates (Loudet *et al.*, 2007; Koprivova *et al.*, 2013). The fact that one of the two major QTLs controlling sulfate reduction, the gene *APR2*, has evolved loss-of-function alleles three times independently, in Central Asia, Czech Republic, and Sweden, was also a striking result (Loudet *et al.*, 2007; Chao *et al.*, 2014).

Studies of natural variation can also inform about the genetic architecture and evolutionary potential of specific traits. For example, the major discoveries driven by differences in ionomes between *A. thaliana* populations were not achieved through genome-wide association mapping (Atwell *et al.*, 2010), but through the use of ionomics to screen for genotypes with contrasted nutrient content for analysis (Lahner *et al.*, 2003; Salt *et al.*, 2008). Approximately 20-fold differences in molybdenum concentration were measured in leaves of 98 *A. thaliana* genotypes, and the genetic analysis of the progeny of two of the most contrasted accessions Col-0 and Ler revealed the role of *Molybdenum Transporter-1* (*MOT1*) (Tomatsu *et al.*, 2007; Baxter *et al.*, 2008). Similarly, tetraploidy was shown to increase potassium content in the progeny of Col-0 and the autotetraploid line Wa-1 (Chao *et al.*, 2013). Such studies demonstrate that heritable variation in nutrient content often results from variants that are (i) large effect mutations since they can be easily dissected in bi-parental progeny and (ii) rare because they do not give a detectable signal in genome-wide association studies (GWAS). This indicates that a plant’s ability to pre-empt resources for improved nutrition can be easily manipulated at the genetic level. Such genetic variants in nutrient uptake can be used to understand population maintenance and plant community formation in a context of nutrient depletion or plant–plant competition. In other words, they provide a valuable resource to understand how molecular mechanisms can contribute to ecological diversification.

For most ionomic traits, however, a contribution to plant growth rate, competitive ability, or stress tolerance has not been established. Accumulation of sodium is one of the rare examples where the ecological relevance of genetic variation for mineral uptake could be documented. An allele of the sodium transporter gene *AtHKT1* was shown to mediate increased Na^+^ concentration in *A. thaliana* genotypes originating from two coastal habitats in Spain and Japan, and was found to co-segregate with salt tolerance (Rus *et al.*, 2006, Baxter *et al.*, 2010). Using distance to sea or to a known saline soil as a quantitative measure, a strong relationship between high leaf Na^+^ and origin in potentially saline-impacted soils was confirmed (Baxter *et al.*, 2010). Recently, the mechanism by which the weak allele of *AtHKT1* confers Na^+^ tolerance has been elucidated (An *et al.*, 2017). High expression of *AtHKT1* in stems strongly limits the allocation of Na^+^ to reproductive tissues and thus confers higher fertility specifically under salt stress (An *et al.*, 2017).

An ecological perspective on functional variation can also allow a more comprehensive description of gene function. For example, the gene *FLC* was named after its typical effect on flowering time: *flc* mutants are considerably earlier flowering in long day-controlled conditions (Michaels and Amasino, 1999). The dissection of natural variation present at this locus in *A. thaliana* uncovered an allelic series conferring a wide range of flowering times and responses to vernalization (Lempe *et al.*, 2005; Shindo *et al.*, 2005; Coustham *et al.*, 2012; Li *et al.*, 2014). Allelic variation at *FLC* orthologs is also responsible for variation in flowering time or duration of flowering in other crucifer species (Albani *et al.*, 2012; Kemi *et al.*, 2013; Baduel *et al.*, 2016). Progressively, however, the specificity of FLC action on flowering time has been questioned. *FLC* variation was associated with the timing of germination (Chiang *et al.*, 2009), raising the possibility that *FLC* acts pleiotropically on multiple phenotypes. Indeed, the genome-wide analysis of FLC-binding sites uncovered several hundred genes, a large proportion of which were involved in response to cold stress (Deng *et al.*, 2011; Mateos *et al.*, 2015, 2017). Several genes involved in cold stress were strongly deregulated in *flc* mutants compared with the *FLC* wild type when plants were exposed to cold, but not at normal growth temperatures, suggesting that FLC has a role in modulating expression of genes conferring tolerance to cold (Mateos *et al.*, 2017). Pleiotropic genes such as *FLC* may respond to the fundamental requirement for ecological pleiotropy in natural environments that are marked by inevitable fluctuations. Indeed, the monitoring of frequency changes in alleles associated with various reproductive and phenological traits in the field within a single natural *A. thaliana* population showed that variants with intermediate levels of pleiotropy contributed the largest adaptive steps (Frachon *et al.*, 2017). This is because selective forces fluctuate across years and seasons, and they are more likely to act consistently on variants controlling more than one phenotype. Natural selection at this particular site thus appeared to have favored variants contributing to both increased tolerance to local warming and increased competitive ability (Frachon *et al.*, 2017). Although it is beyond the scope of this review to enumerate all molecular functions whose ecological role remains to be fully determined, the examples given by plant nutrition or the pleiotropic effects of *FLC* illustrate how placing molecular functions within the context of ecological strategies helps identify genes and their ultimate role in natural conditions.

## Towards resolving ecological questions with a genetically tractable plant system

Conversely, the diverse ecological strategies co-segregating in *A. thaliana* provides a unique system to address, at the genetic and molecular level, those questions that are key to ecologists. We illustrate this idea below with a pressing question in current ecological research: the impact of climate change.

In today’s ecological research, discerning the mechanisms behind ecosystem responses to climate change is a central theme (Reed *et al.*, 2012; Ruppert *et al.*, 2015). Extended periods of high temperature and net declines in soil moisture are expected in many regions (IPCC, 2013). Intraspecific variation in functional traits associated with resource-use efﬁciency and stress tolerance may help understand the determinants of species growth and survival under climate change (Aspinwall *et al.*, 2013).

A first consequence of increased climatic unpredictability is that ecological shifts towards increasingly ruderal strategies will be promoted. The study of flowering time variation in *A. thaliana* has demonstrated that species are not limited in the number of mutations that can promote accelerated flowering (Mendez-Vigo *et al.*, 2011; Sánchez-Bermejo *et al.*, 2012; Hepworth and Dean, 2015; Whittaker and Dean, 2017): species will therefore have ample opportunities to adapt to drought by advancing their transition to flowering (Franks *et al.*, 2007). As a matter of fact, earlier flowering seems to be globally under selection (Munguía-Rosas *et al.*, 2011).

However, adapting the timing of major life history transitions will probably not suffice. As water is a paramount factor in determining both the distribution and the productivity of plant species, drought stress responses will be increasingly critical for species assemblages in most environments (Volaire, 2018). Through manipulative experiments and data fusion approaches, ecologists have learned what they may basically expect for ecosystem dynamics: individual-level responses are followed by species reordering within communities, and ﬁnally by species losses and immigration (Smith *et al.*, 2009). These observations lack a generic understanding of individual-level responses, which are typically initiated at the molecular level, and then cascade upwards to affect plant individuals’ physiology and growth (Chaves *et al.*, 2003; Avolio *et al.*, 2018). Unfortunately, detecting and linking cascading stress responses across levels of biological organization is highly challenging (Meyer *et al.*, 2014; Lovell *et al.*, 2016), partly due to the use of different conceptual frameworks and terminologies across the different disciplines and scales (Volaire, 2018). Although ecologists have grown increasingly interested in linking molecular drought responses with physiological data from plant individuals (Lovell *et al.*, 2016; Hoffman and Smith, 2018), very few studies up to now have examined the link between different levels of biological organization in plant water stress responses (Avolio *et al.*, 2018). Besides the research challenges described above, this is partly due to a reluctance of ecologists to include an ecological outlier such as the ruderal *A. thaliana*.

Ecologists nevertheless increasingly acknowledge that an understanding of gene expression is a critical hurdle for dissecting stress response mechanisms (Hoffman and Smith, 2018). Many studies focusing on drought ecology have been conducted in perennial grasses such as *Andropogon gerardii, Sorghastrum nutans*, or *Panicum virgatum* (Hoover *et al.*, 2014). Agronomic studies have been mostly conducted on domesticated, annual grasses such as durum wheat (*Triticum turgidum*) or barley (*Hordeum vulgare*). In light of the comparatively high ecological diversification reviewed above, one can argue that the annual forb *A. thaliana* could efficiently complement these studies. Some recent, interdisciplinary attempts have exemplified how such a diverse species could help us understand the mechanisms and ecological trade-offs of stress responses. Combining a characterization of genetic variation in drought stress resistance with current and future climate envelopes revealed the enormous adaptive potential of *A. thaliana* in the face of climate change (Fournier-Level *et al.*, 2016; Exposito-Alonso *et al.*, 2018*a*). It also documented the genetics of this potential (Exposito-Alonso *et al.* 2018*b*, *c*; Fournier-Level *et al.*, 2016). Among European genotypes, it is predicted that those originating from Northern and Southern latitudes will be able to adapt in the new climate, due to their higher drought resistance as well as the genetic variability of the populations (Exposito-Alonso *et al.*, 2018*c*). In fact, in *A. thaliana*, it is possible to perform experiments quantifying the impact of selection in populations faced with increasingly realistic scenarios of global climate change, where exposure to drought stress, average temperature, or increased frequency of major disturbance set new limits on plant plasticity (Exposito-Alonso *et al.*, 2018*b*). To enhance the comparability of drought studies across model species and disciplines, drought regimes (e.g. frequency and intensity) should also be characterized with standard methods in the species (Vicca *et al.*, 2012; Ruppert *et al.*, 2015), and diagnostic experimental procedures should be adopted to identify the ecological mechanisms promoting drought resistance (Gilbert and Medina, 2016).

As an undomesticated species, *A. thaliana* has been subject to a complex suite of environmental challenges over the course of its evolutionary history (see Koch, 2019), which promoted a diversification in ecological strategy. Its amenability to genetic approaches (e.g. seed stocks, mapping populations, mutant collections, or GWAS panels) can greatly facilitate trait analysis and reveal which functional traits or trait combinations are sufficient to promote shifts in ecological strategies. For example, dissecting how the plant combines tolerance to multiple stresses, whilst at the same time fine-tuning the balance between defense, growth, and productivity, is of great importance for interpreting the dynamics of plant communities (Bechtold *et al.*, 2018). Knowing which genes contribute to unobservable traits underpinning key aspects of ecological strategy can also improve the ecological classification of species. Indeed, they provide measurable proxies for traits that are difficult to measure but make important contributions to dimenstions of the ecological strategy that leaf traits and the CSR strategy scheme do not fully recapitulate.

## Conclusion and outlook

The high natural variation and the unrivaled genomic resources of *A. thaliana* are great assets in understanding pressing questions in contemporary plant ecology but also to dissect gene function comprehensively, from the molecular to the community level. This review has assembled recent conceptual and methodological developments that show how this field is advancing. The advent of new sequencing technologies has increasingly digitalized observations both in the lab and in the field. To enhance our interpretation of these data, links between specific genes and the evolution of novel ecological preferences must be established. The recently published indication that variation in CSR positioning contributes to local adaptation in *A. thaliana* already suggests that variation in global ecological strategy is both heritable and relevant for understanding plant performance in diverse parts of the species range (Vasseur *et al.* 2018*b*). Yet, variation in CSR positioning also depends on the environment in which CSR-indicative traits are measured (Vasseur *et al.* 2018*b*). Several key challenges remain to be addressed such as the following: (i) To what extent do intraspecific changes in CSR positioning translate into changes in competitive ability, stress resistance, or tolerance to disturbance? (ii) How many traits in a trait syndrome are sufficient to initiate significant ecological shifts? (iii) What is the importance of plasticity in shifting ecological strategies? (iv) Which gene or gene activity can be used as proxy to quantify ecological dimensions that are not correctly summarized in global strategy schemes? Answering these questions in *A. thaliana* will pave the way for bridging ecology and molecular biology in Plant Sciences.

## References

[CIT0001] Ågren J, Oakley CG, Lundemo S, Schemske DW 2017 Adaptive divergence in flowering time among natural populations of *Arabidopsis thaliana*: estimates of selection and QTL mapping. Evolution 71, 550–564. 27859214 10.1111/evo.13126

[CIT0002] Agren J, Schemske DW 2012 Reciprocal transplants demonstrate strong adaptive differentiation of the model organism *Arabidopsis thaliana* in its native range. New Phytologist 194, 1112–1122. 22432639 10.1111/j.1469-8137.2012.04112.x

[CIT0003] Akiyama R, Ågren J 2014 Conflicting selection on the timing of germination in a natural population of *Arabidopsis thaliana*. Journal of Evolutionary Biology 27, 193–199. 24329869 10.1111/jeb.12293

[CIT0004] Albani MC, Castaings L, Wötzel S, Mateos JL, Wunder J, Wang R, Reymond M, Coupland G 2012 PEP1 of *Arabis alpina* is encoded by two overlapping genes that contribute to natural genetic variation in perennial flowering. PLoS Genetics 8, e1003130. 23284298 10.1371/journal.pgen.1003130 PMC3527215

[CIT0005] Alonso-Blanco C, Aarts MG, Bentsink L, Keurentjes JJ, Reymond M, Vreugdenhil D, Koornneef M 2009 What has natural variation taught us about plant development, physiology, and adaptation? The Plant Cell 21, 1877–1896. 19574434 10.1105/tpc.109.068114 PMC2729614

[CIT0006] An D, Chen JG, Gao YQ, et al 2017 AtHKT1 drives adaptation of *Arabidopsis thaliana* to salinity by reducing floral sodium content. PLoS Genetics 13, e1007086. 29084222 10.1371/journal.pgen.1007086 PMC5679648

[CIT0007] Aspinwall MJ, Lowry DB, Taylor SH, Juenger TE, Hawkes CV, Johnson MV, Kiniry JR, Fay PA 2013 Genotypic variation in traits linked to climate and aboveground productivity in a widespread C_4_ grass: evidence for a functional trait syndrome. New Phytologist 199, 966–980. 23701159 10.1111/nph.12341

[CIT0008] Astuti G, Ciccarelli D, Roma-Marzio F, Trinco A, Peruzzi L 2018 Narrow endemic species *Bellevalia webbiana* shows significant intraspecific variation in tertiary CSR strategy. Plant Biosystems (in press).

[CIT0009] Atwell S, Huang YS, Vilhjálmsson BJ, et al 2010 Genome-wide association study of 107 phenotypes in *Arabidopsis thaliana* inbred lines. Nature 465, 627–631. 20336072 10.1038/nature08800 PMC3023908

[CIT0010] Austen EJ, Rowe L, Stinchcombe JR, Forrest JRK 2017 Explaining the apparent paradox of persistent selection for early flowering. New Phytologist 215, 929–934. 28418161 10.1111/nph.14580

[CIT0011] Avolio ML, Hoffman AM, Smith MD 2018 Linking gene regulation, physiology, and plant biomass allocation in *Andropogon gerardii* in response to drought. Plant Ecology 219, 1–15.

[CIT0012] Bac-Molenaar JA, Granier C, Keurentjes JJ, Vreugdenhil D 2016 Genome-wide association mapping of time-dependent growth responses to moderate drought stress in *Arabidopsis*. Plant, Cell & Environment 39, 88–102. 10.1111/pce.12595 26138664

[CIT0013] Baduel P, Arnold B, Weisman CM, Hunter B, Bomblies K 2016 Habitat-associated life history and stress-tolerance variation in *Arabidopsis arenosa*. Plant Physiology 171, 437–451. 26941193 10.1104/pp.15.01875 PMC4854687

[CIT0014] Baron E, Richirt J, Villoutreix R, Amsellem L 2015 The genetics of intra- and interspecific competitive response and effect in a local population of an annual plant species. Functional Ecology 29, 1361–1370.

[CIT0015] Bechtold U, Ferguson JN, Mullineaux PM 2018 To defend or to grow: lessons from *Arabidopsis* C24. Journal of Experimental Botany 69, 2809–2821. 29562306 10.1093/jxb/ery106

[CIT0016] Baxter I, Brazelton JN, Yu D, et al 2010 A coastal cline in sodium accumulation in *Arabidopsis thaliana* is driven by natural variation of the sodium transporter AtHKT1;1. PLoS Genetics 6, e1001193. 21085628 10.1371/journal.pgen.1001193 PMC2978683

[CIT0017] Baxter I, Muthukumar B, Park HC, et al 2008 Variation in molybdenum content across broadly distributed populations of *Arabidopsis thaliana* is controlled by a mitochondrial molybdenum transporter (MOT1). PLoS Genetics 4, e1000004. 18454190 10.1371/journal.pgen.1000004 PMC2265440

[CIT0018] Bentsink L, Yuan K, Koornneef M, Vreugdenhil D 2003 The genetics of phytate and phosphate accumulation in seeds and leaves of *Arabidopsis thaliana*, using natural variation. Theoretical and Applied Genetics 106, 1234–1243. 12748774 10.1007/s00122-002-1177-9

[CIT0019] Bilton MC, Whitlock R, Grime JP, Marion G, Pakeman RJ 2010 Intraspecific trait variation in grassland plant species reveals fine-scale strategy trade-offs and size differentiation that underpins performance in ecological communities. Botany 88, 939–952.

[CIT0020] Bomblies K, Yant L, Laitinen RA, Kim ST, Hollister JD, Warthmann N, Fitz J, Weigel D 2010 Local-scale patterns of genetic variability, outcrossing, and spatial structure in natural stands of *Arabidopsis thaliana*. PLoS Genetics 6, e1000890. 20361058 10.1371/journal.pgen.1000890 PMC2845663

[CIT0021] Brachi B, Aimé C, Glorieux C, Cuguen J, Roux F 2012 Adaptive value of phenological traits in stressful environments: predictions based on seed production and laboratory natural selection. PLoS One 7, e32069. 22403624 10.1371/journal.pone.0032069 PMC3293886

[CIT0022] Brachi B, Faure N, Horton M, Flahauw E, Vazquez A, Nordborg M, Bergelson J, Cuguen J, Roux F 2010 Linkage and association mapping of *Arabidopsis thaliana* flowering time in nature. PLoS Genetics 6, e1000940. 20463887 10.1371/journal.pgen.1000940 PMC2865524

[CIT0023] Burghardt LT, Metcalf CJ, Wilczek AM, Schmitt J, Donohue K 2015 Modeling the influence of genetic and environmental variation on the expression of plant life cycles across landscapes. The American Naturalist 185, 212–227. 10.1086/679439 25616140

[CIT0024] Campitelli BE, Des Marais DL, Juenger TE 2016 Ecological interactions and the fitness effect of water-use efficiency: competition and drought alter the impact of natural MPK12 alleles in *Arabidopsis*. Ecology Letters 19, 424–434. 26868103 10.1111/ele.12575

[CIT0025] Chao DY, Baraniecka P, Danku J, Koprivova A, Lahner B, Luo H, Yakubova E, Dilkes B, Kopriva S, Salt DE 2014 Variation in sulfur and selenium accumulation is controlled by naturally occurring isoforms of the key sulfur assimilation enzyme ADENOSINE 5'-PHOSPHOSULFATE REDUCTASE2 across the *Arabidopsis* species range. Plant Physiology 166, 1593–1608. 25245030 10.1104/pp.114.247825 PMC4226352

[CIT0026] Chao DY, Dilkes B, Luo H, Douglas A, Yakubova E, Lahner B, Salt DE 2013 Polyploids exhibit higher potassium uptake and salinity tolerance in *Arabidopsis*. Science 341, 658–659. 23887874 10.1126/science.1240561 PMC4018534

[CIT0027] Chaves MM, Maroco JP, Pereira JS 2003 Understanding plant responses to drought—from genes to the whole plant. Functional Plant Biology 30, 239–264. 10.1071/FP02076 32689007

[CIT0028] Chiang GCK, Barua D, Dittmar E, Kramer EM, de Casas RR, Donohue K 2013 Pleiotropy in the wild: the dormancy gene DOG1 exerts cascading control on life cycles. Evolution 67, 883–893. 23461337 10.1111/j.1558-5646.2012.01828.x

[CIT0029] Chiang GCK, Barua D, Dittmar E, Kramer EM, Amasino RM, Donohue K 2009 Major flowering time gene, *FLOWERING LOCUS C*, regulates seed germination in *Arabidopsis thaliana*. Proceedings of the National Academy of Sciences, USA 106, 11661–11666. 10.1073/pnas.0901367106 PMC2710639 19564609

[CIT0030] Clauss MJ, Koch MA 2006 Poorly known relatives of *Arabidopsis thaliana*. Trends in Plant Science 11, 449–459. 16893672 10.1016/j.tplants.2006.07.005

[CIT0031] Clauw P, Coppens F, Korte A, et al 2016 Leaf growth response to mild drought: natural variation in *Arabidopsis* sheds light on trait architecture. The Plant Cell 28, 2417–2434. 27729396 10.1105/tpc.16.00483 PMC5134983

[CIT0032] Coustham V, Li P, Strange A, Lister C, Song J, Dean C 2012 Quantitative modulation of polycomb silencing underlies natural variation in vernalization. Science 337, 584–587. 22798408 10.1126/science.1221881

[CIT0033] Davila Olivas NH, Kruijer W, Gort G, Wijnen CL, van Loon JJ, Dicke M 2017 Genome-wide association analysis reveals distinct genetic architectures for single and combined stress responses in *Arabidopsis thaliana*. New Phytologist 213, 838–851. 27604707 10.1111/nph.14165 PMC5217058

[CIT0034] Debieu M, Tang C, Stich B, Sikosek T, Effgen S, Josephs E, Schmitt J, Nordborg M, Koornneef M, de Meaux J 2013 Co-variation between seed dormancy, growth rate and flowering time changes with latitude in *Arabidopsis thaliana*. PLoS One 8, e61075. 23717385 10.1371/journal.pone.0061075 PMC3662791

[CIT0035] Deng W, Ying H, Helliwell CA, Taylor JM, Peacock WJ, Dennis ES 2011 FLOWERING LOCUS C (FLC) regulates development pathways throughout the life cycle of *Arabidopsis*. Proceedings of the National Academy of Sciences, USA 108, 6680–6685. 10.1073/pnas.1103175108 PMC3081018 21464308

[CIT0036] Des Marais DL, McKay JK, Richards JH, Sen S, Wayne T, Juenger TE 2012 Physiological genomics of response to soil drying in diverse *Arabidopsis* accessions. The Plant Cell 24, 893–914. 22408074 10.1105/tpc.112.096180 PMC3336118

[CIT0037] Des Roches S, Post DM, Turley NE, Bailey JK, Hendry AP, Kinnison MT, Schweitzer JA, Palkovacs EP 2018 The ecological importance of intraspecific variation. Nature Ecology & Evolution 2, 57–64. 29203921 10.1038/s41559-017-0402-5

[CIT0038] Díaz S, Kattge J, Cornelissen JH, et al 2016 The global spectrum of plant form and function. Nature 529, 167–171. 26700811 10.1038/nature16489

[CIT0039] Dittberner H, Korte A, Mettler-Altmann T, Weber APM, Monroe G, de Meaux J 2018 Natural variation in stomata size contributes to the local adaptation of water-use efficiency in *Arabidopsis thaliana*. Molecular Ecology 27, 4052–4065. 30118161 10.1111/mec.14838 PMC7611081

[CIT0040] Donohue K 2002 Germination timing influences natural selection on life-history characters in *Arabidopsis thaliana*. Ecology 83, 1006–1016.

[CIT0041] Donohue K, Dorn L, Griffith C, Kim E, Aguilera A, Polisetty CR, Schmitt J 2005 Niche construction through germination cueing: life-history responses to timing of germination in *Arabidopsis thaliana*. Evolution 59, 771–785. 15926688

[CIT0042] Durvasula A, Fulgione A, Gutaker RM, et al 2017 African genomes illuminate the early history and transition to selfing in *Arabidopsis thaliana*. Proceedings of the National Academy of Sciences, USA 114, 5213–5218. 10.1073/pnas.1616736114 PMC5441814 28473417

[CIT0043] Easlon HM, Nemali KS, Richards JH, Hanson DT, Juenger TE, McKay JK 2014 The physiological basis for genetic variation in water use efficiency and carbon isotope composition in *Arabidopsis thaliana*. Photosynthesis Research 119, 119–129. 23893317 10.1007/s11120-013-9891-5 PMC3889294

[CIT0044] El-Soda M, Kruijer W, Malosetti M, Koornneef M, Aarts MG 2015 Quantitative trait loci and candidate genes underlying genotype by environment interaction in the response of *Arabidopsis thaliana* to drought. Plant, Cell & Environment 38, 585–599. 10.1111/pce.12418 25074022

[CIT0045] Exposito-Alonso M, Becker C, Schuenemann VJ, et al 2018a. The rate and potential relevance of new mutations in a colonizing plant lineage. PLoS Genetics 14, e1007155. 29432421 10.1371/journal.pgen.1007155 PMC5825158

[CIT0046] Exposito-Alonso M, Brennan A, Alonso-Blanco C, Picó FX 2018b. Spatio-temporal variation in fitness responses to contrasting environments in *Arabidopsis thaliana*. Evolution 72, 1570–1586. 10.1111/evo.13508 29947421

[CIT0047] Exposito-Alonso M, Vasseur F, Ding W, Wang G, Burbano HA, Weigel D 2018c. Genomic basis and evolutionary potential for extreme drought adaptation in *Arabidopsis thaliana*. Nature Ecology & Evolution 2, 352–358. 29255303 10.1038/s41559-017-0423-0 PMC5777624

[CIT0048] Footitt S, Huang Z, Clay HA, Mead A, Finch-Savage WE 2013 Temperature, light and nitrate sensing coordinate *Arabidopsis* seed dormancy cycling, resulting in winter and summer annual phenotypes. The Plant Journal 74, 1003–1015. 23590427 10.1111/tpj.12186 PMC3764396

[CIT0049] Fournier-Level A, Korte A, Cooper MD, Nordborg M, Schmitt J, Wilczek AM 2011 A map of local adaptation in *Arabidopsis thaliana*. Science 334, 86–89. 21980109 10.1126/science.1209271

[CIT0050] Fournier-Level A Perry EO, Wang JA, Braun PT, Migneault A, Cooper MD, Metcalf CJE, Schmitt J 2016 Dynamics of seasonal adaptation in *A. thaliana*. Proceedings of the National Academy of Sciences, USA 113, E2812–E2821. 10.1073/pnas.1517456113 PMC4878477 27140640

[CIT0051] Fournier-Level A, Wilczek AM, Cooper MD, et al 2013 Paths to selection on life history loci in different natural environments across the native range of *Arabidopsis thaliana*. Molecular Ecology 22, 3552–3566. 23506537 10.1111/mec.12285

[CIT0052] Franks SJ 2011 Plasticity and evolution in drought avoidance and escape in the annual plant *Brassica rapa*. New Phytologist 190, 249–257. 21210818 10.1111/j.1469-8137.2010.03603.x

[CIT0053] Franks SJ, Sim S, Weis AE 2007 Rapid evolution of flowering time by annual plant in response to a climate fluctuation. Proceedings of the National Academy of Sciences, USA 104, 1278–1282. 10.1073/pnas.0608379104 PMC1783115 17220273

[CIT0054] Frachon L, Libourel C, Villoutreix R, et al 2017 Intermediate degrees of synergistic pleiotropy drive adaptive evolution in ecological time. Nature Ecology & Evolution 1, 1551–1561. 29185515 10.1038/s41559-017-0297-1

[CIT0055] **1001 Genomes Consortium** 2016 1,135 genomes reveal the global pattern of polymorphism in *Arabidopsis thaliana*. Cell 166, 481–491. 27293186 10.1016/j.cell.2016.05.063 PMC4949382

[CIT0056] Gilbert ME, Medina V 2016 Drought adaptation mechanisms should guide experimental design. Trends in Plant Science 21, 639–647. 27090148 10.1016/j.tplants.2016.03.003

[CIT0057] Glander S, He F, Schmitz G, Witten A, Telschow A, de Meaux J 2018 Assortment of flowering time and immunity alleles in natural *Arabidopsis thaliana* populations suggests immunity and vegetative lifespan strategies coevolve. Genome Biology and Evolution 10, 2278–2291. 30215800 10.1093/gbe/evy124 PMC6133262

[CIT0058] Grime JP 1974 Vegetation classification by reference to strategies. Nature 250, 26–31.

[CIT0059] Grime JP 1977 Evidence for the existence of three primary strategies in plants and its relevance to ecological and evolutionary theory. The American Naturalist 111, 1169–1194.

[CIT0060] Hancock AM, Brachi B, Faure N, Horton MW, Jarymowycz LB, Sperone FG, Toomajian C, Roux F, Bergelson J 2011 Adaptation to climate across the *Arabidopsis thaliana* genome. Science 334, 83–86. 21980108 10.1126/science.1209244

[CIT0061] Harada H, Kuromori T, Hirayama T, Shinozaki K, Leigh RA 2004 Quantitative trait loci analysis of nitrate storage in *Arabidopsis* leading to an investigation of the contribution of the anion channel gene, AtCLC-c, to variation in nitrate levels. Journal of Experimental Botany 55, 2005–2014. 15310822 10.1093/jxb/erh224

[CIT0062] He F, Kang D, Ren Y, Qu LJ, Zhen Y, Gu H 2007 Genetic diversity of the natural populations of *Arabidopsis thaliana* in China. Heredity 99, 423–431. 17593944 10.1038/sj.hdy.6801020

[CIT0063] He H, de Souza Vidigal D, Snoek LB, Schnabel S, Nijveen H, Hilhorst H, Bentsink L 2014 Interaction between parental environment and genotype affects plant and seed performance in *Arabidopsis*. Journal of Experimental Botany 65, 6603–6615. 25240065 10.1093/jxb/eru378 PMC4246189

[CIT0064] Hepworth J, Dean C 2015 Flowering Locus C’s lessons: conserved chromatin switches underpinning developmental timing and adaptation. Plant Physiology 168, 1237–1245. 26149571 10.1104/pp.15.00496 PMC4528751

[CIT0065] Hoffman AM, Smith MD 2018 Gene expression differs in codominant prairie grasses under drought. Molecular Ecology Resources 18, 334–346. 29098789 10.1111/1755-0998.12733

[CIT0066] Hoffmann MH 2005 Evolution of the realized climatic niche in the genus *Arabidopsis* (Brassicaceae). Evolution 59, 1425–1436. 16153029

[CIT0067] Hoffmann MH 2002 Biogeography of *Arabidopsis thaliana* (L.) Heynh. (Brassicaceae). Journal of Biogeography 29, 125–134.

[CIT0068] Hoover DL, Knapp AK, Smith MD 2014 Resistance and resilience of a grassland ecosystem to climate extremes. Ecology 95:2646–2656.

[CIT0069] Hu J, Lei L, de Meaux J 2017 Temporal fitness fluctuations in experimental *Arabidopsis thaliana* populations. PLoS One 12, e0178990. 28604796 10.1371/journal.pone.0178990 PMC5467858

[CIT0070] **IPCC** 2013 Climate Change 2013: the physical science basis. Working Group I Contribution to the Intergovernmental Panel on Climate Change Fifth Assessment Report. Cambridge, UK: Cambridge University Press.

[CIT0071] Johanson U, West J, Lister C, Michaels S, Amasino R, Dean C 2000 Molecular analysis of FRIGIDA, a major determinant of natural variation in *Arabidopsis* flowering time. Science 290, 344–347. 11030654 10.1126/science.290.5490.344

[CIT0072] Kasulin L, Rowan BA, León RJC, Schuenemann VJ, Weigel D, Botto JF 2017 A single haplotype hyposensitive to light and requiring strong vernalization dominates *Arabidopsis thaliana* populations in Patagonia, Argentina. Molecular Ecology 26, 3389–3404. 28316114 10.1111/mec.14107

[CIT0073] Kemi U, Niittyvuopio A, Toivainen T, Pasanen A, Quilot-Turion B, Holm K, Lagercrantz U, Savolainen O, Kuittinen H 2013 Role of vernalization and of duplicated FLOWERING LOCUS C in the perennial *Arabidopsis lyrata*. New Phytologist 197, 323–335. 23106477 10.1111/j.1469-8137.2012.04378.x

[CIT0074] Kerdaffrec E, Filiault DL, Korte A, Sasaki E, Nizhynska V, Seren Ü, Nordborg M 2016 Multiple alleles at a single locus control seed dormancy in Swedish *Arabidopsis*. eLife 5, e22502. 27966430 10.7554/eLife.22502 PMC5226650

[CIT0075] Kesari R, Lasky JR, Villamor JG, Des Marais DL, Chen Y-JC, Liu T-W, Lin W, Juenger TE, Verslues PE 2012 Intron-mediated alternative splicing of *Arabidopsis* P5CS1 and its association with natural variation in proline and climate adaptation. Proceedings of the National Academy of Sciences, USA 109, 9197–9202. 10.1073/pnas.1203433109 PMC3384178 22615385

[CIT0076] Kiefer C, Severing E, Karl R, Bergonzi S, Koch M, Tresch A, Coupland G 2017 Divergence of annual and perennial species in the Brassicaceae and the contribution of cis-acting variation at FLC orthologues. Molecular Ecology 26, 3437–3457. 28261921 10.1111/mec.14084 PMC5485006

[CIT0077] Koch MA 2019 The plant model system Arabidopsis set into an evolutionary, systematic, and spatio-temporal context. Journal of Experimental Botany 70, 55–67. 30260404 10.1093/jxb/ery340

[CIT0078] Koornneef M, Alonso-Blanco C, Vreugdenhil D 2004 Naturally occurring genetic variation in *Arabidopsis thaliana*. Annual Review of Plant Biology 55, 141–172. 10.1146/annurev.arplant.55.031903.141605 15377217

[CIT0079] Koprivova A, Giovannetti M, Baraniecka P, Lee BR, Grondin C, Loudet O, Kopriva S 2013 Natural variation in the ATPS1 isoform of ATP sulfurylase contributes to the control of sulfate levels in *Arabidopsis*. Plant Physiology 163, 1133–1141. 24027241 10.1104/pp.113.225748 PMC3813639

[CIT0081] Kraft NJ, Valencia R, Ackerly DD 2008 Functional traits and niche-based tree community assembly in an Amazonian forest. Science 322, 580–582. 18948539 10.1126/science.1160662

[CIT0082] Kronholm I, Picó FX, Alonso-Blanco C, Goudet J, de Meaux J 2012 Genetic basis of adaptation in *Arabidopsis thaliana*: local adaptation at the seed dormancy QTL DOG1. Evolution 66, 2287–2302. 22759302 10.1111/j.1558-5646.2012.01590.x

[CIT0083] Lahner B, Gong J, Mahmoudian M, et al 2003 Genomic scale profiling of nutrient and trace elements in *Arabidopsis thaliana*. Nature Biotechnology 21, 1215–1221. 10.1038/nbt865 12949535

[CIT0084] Lasky JR, Des Marais DL, Lowry DB, Povolotskaya I, McKay JK, Richards JH, Keitt TH, Juenger TE 2014 Natural variation in abiotic stress responsive gene expression and local adaptation to climate in *Arabidopsis thaliana*. Molecular Biology and Evolution 31, 2283–2296. 24850899 10.1093/molbev/msu170 PMC4137704

[CIT0085] Le Corre V 2005 Variation at two flowering time genes within and among populations of *Arabidopsis thaliana*: comparison with markers and traits. Molecular Ecology 14, 4181–4192. 16262868 10.1111/j.1365-294X.2005.02722.x

[CIT0086] Lempe J, Balasubramanian S, Sureshkumar S, Singh A, Schmid M, Weigel D 2005 Diversity of flowering responses in wild *Arabidopsis thaliana* strains. PLoS Genetics 1, 109–118. 16103920 10.1371/journal.pgen.0010006 PMC1183525

[CIT0087] Li P, Filiault D, Box MS, et al 2014 Multiple FLC haplotypes defined by independent cis-regulatory variation underpin life history diversity in *Arabidopsis thaliana*. Genes & Development 28, 1635–1640. 25035417 10.1101/gad.245993.114 PMC4117938

[CIT0088] Loudet O, Chaillou S, Merigout P, Talbotec J, Daniel-Vedele F 2003 Quantitative trait loci analysis of nitrogen use efficiency in *Arabidopsis*. Plant Physiology 131, 345–358. 12529542 10.1104/pp.102.010785 PMC166814

[CIT0089] Loudet O, Saliba-Colombani V, Camilleri C, Calenge F, Gaudon V, Koprivova A, North KA, Kopriva S, Daniel-Vedele F 2007 Natural variation for sulfate content in *Arabidopsis thaliana* is highly controlled by APR2. Nature Genetics 39, 896–900. 17589509 10.1038/ng2050

[CIT0090] Lovell JT, Shakirov EV, Schwartz S, et al 2016 Promises and challenges of eco-physiological genomics in the field: tests of drought responses in switchgrass. Plant Physiology 172, 734–748. 27246097 10.1104/pp.16.00545 PMC5047078

[CIT0091] Lovell JT, Juenger TE, Michaels SD, Lasky JR, Platt A, Richards JH, Yu X, Easlon HM, Sen S, McKay JK 2013 Pleiotropy of FRIGIDA enhances the potential for multivariate adaptation. Proceedings of the Royal Society B: Biological Sciences 280, 1763. 10.1098/rspb.2013.1043 PMC3774242 23698015

[CIT0092] Marcer A, Vidigal DS, James PMA, Fortin MJ, Méndez-Vigo B, Hilhorst HWM, Bentsink L, Alonso-Blanco C, Picó FX 2018 Temperature fine-tunes Mediterranean *Arabidopsis thaliana* life-cycle phenology geographically. Plant Biology 20 (Suppl 1), 148–156. 28241389 10.1111/plb.12558

[CIT0093] Marchadier E, Hanemian M, Tisne S, Bach L, Bazakos C, Gilbault E, Haddadi P, Virlouvet L, Loudet O 2018 The complex genetic architecture of shoot growth natural variation in *Arabidopsis thaliana*. BioRxiv 354738. [Preprint]. 10.1371/journal.pgen.1007954 PMC6476473 31009456

[CIT0094] Masclaux F, Hammond RL, Meunier J, Gouhier-Darimont C, Keller L, Reymond P 2010 Competitive ability not kinship affects growth of *Arabidopsis thaliana* accessions. New Phytologist 185, 322–331. 19886895 10.1111/j.1469-8137.2009.03057.x

[CIT0095] Mateos JL, Madrigal P, Tsuda K, Rawat V, Richter R, Romera-Branchat M, Fornara F, Schneeberger K, Krajewski P, Coupland G 2015 Combinatorial activities of SHORT VEGETATIVE PHASE and FLOWERING LOCUS C define distinct modes of flowering regulation in *Arabidopsis*. Genome Biology 16, 31. 25853185 10.1186/s13059-015-0597-1 PMC4378019

[CIT0096] Mateos JL, Tilmes V, Madrigal P, Severing E, Richter R, Rijkenberg CWM, Krajewski P, Coupland G 2017 Divergence of regulatory networks governed by the orthologous transcription factors FLC and PEP1 in Brassicaceae species. Proceedings of the National Academy of Sciences, USA 114, E11037–E11046. 10.1073/pnas.1618075114 PMC5754749 29203652

[CIT0097] May RL, Warner S, Wingler A 2017 Classification of intra-specific variation in plant functional strategies reveals adaptation to climate. Annals of Botany 119, 1343–1352. 28369157 10.1093/aob/mcx031 PMC5604582

[CIT0098] McKay JK, Richards JH, Mitchell-Olds T 2003 Genetics of drought adaptation in *Arabidopsis thaliana*: I. Pleiotropy contributes to genetic correlations among ecological traits. Molecular Ecology 12, 1137–1151. 12694278 10.1046/j.1365-294x.2003.01833.x

[CIT0099] Méndez-Vigo B, Picó FX, Ramiro M, Martínez-Zapater JM, Alonso-Blanco C 2011 Altitudinal and climatic adaptation is mediated by flowering traits and FRI, FLC, and PHYC genes in *Arabidopsis*. Plant Physiology 157, 1942–1955. 21988878 10.1104/pp.111.183426 PMC3327218

[CIT0100] Meyer E, Aspinwall MJ, Lowry DB, Palacio-Mejía JD, Logan TL, Fay PA, Juenger TE 2014 Integrating transcriptional, metabolomic, and physiological responses to drought stress and recovery in switchgrass (*Panicum virgatum* L.). BMC Genomics 15, 527. 24964784 10.1186/1471-2164-15-527 PMC4122788

[CIT0101] Michaels SD, Amasino RM 1999 FLOWERING LOCUS C encodes a novel MADS domain protein that acts as a repressor of flowering. The Plant Cell 11, 949–956. 10330478 10.1105/tpc.11.5.949 PMC144226

[CIT0102] Mojica JP, Mullen J, Lovell JT, Monroe JG, Paul JR, Oakley CG, McKay JK 2016 Genetics of water use physiology in locally adapted *Arabidopsis thaliana*. Plant Science 251, 12–22. 27593459 10.1016/j.plantsci.2016.03.015

[CIT0103] Montesinos-Navarro A, Wig J, Pico FX, Tonsor SJ 2011 *Arabidopsis thaliana* populations show clinal variation in a climatic gradient associated with altitude. New Phytologist 189, 282–294. 20880224 10.1111/j.1469-8137.2010.03479.x

[CIT0104] Morrison GD, Linder CR 2014 Association mapping of germination traits in *Arabidopsis thaliana* under light and nutrient treatments: searching for G×E effects. G3 4, 1465–1478. 24902604 10.1534/g3.114.012427 PMC4132177

[CIT0105] Munguía-Rosas MA, Ollerton J, Parra-Tabla V, De-Nova JA 2011 Meta-analysis of phenotypic selection on flowering phenology suggests that early flowering plants are favoured. Ecology Letters 14, 511–521. 21332621 10.1111/j.1461-0248.2011.01601.x

[CIT0106] Muñoz-Parra E, Pelagio-Flores R, Raya-González J, Salmerón-Barrera G, Ruiz-Herrera LF, Valencia-Cantero E, López-Bucio J 2017 Plant–plant interactions influence developmental phase transitions, grain productivity and root system architecture in *Arabidopsis* via auxin and PFT1/MED25 signalling. Plant, Cell & Environment 40, 1887–1899. 10.1111/pce.12993 28556372

[CIT0107] Novikova PY, Hohmann N, Nizhynska V, et al 2016 Sequencing of the genus *Arabidopsis* identifies a complex history of nonbifurcating speciation and abundant trans-specific polymorphism. Nature Genetics 48, 1077–1082. 27428747 10.1038/ng.3617

[CIT0108] Picó FX 2012 Demographic fate of *Arabidopsis thaliana* cohorts of autumn- and spring-germinated plants along an altitudinal gradient. Journal of Ecology 100, 1009–1018.

[CIT0109] Picó FX, Méndez-Vigo B, Martínez-Zapater JM, Alonso-Blanco C 2008 Natural genetic variation of *Arabidopsis thaliana* is geographically structured in the Iberian peninsula. Genetics 180, 1009–1021. 18716334 10.1534/genetics.108.089581 PMC2567352

[CIT0110] Pierce S, Negreiros D, Cerabolini BEL, et al 2017 A global method for calculating plant CSR ecological strategies applied across biomes world-wide. Functional Ecology 31, 444–457.

[CIT0111] Postma FM, Ågren J 2015 Maternal environment affects the genetic basis of seed dormancy in *Arabidopsis thaliana*. Molecular Ecology 24, 785–797. 25640699 10.1111/mec.13061

[CIT0112] Postma FM, Ågren J 2016 Early life stages contribute strongly to local adaptation in *Arabidopsis thaliana*. Proceedings of the National Academy of Sciences, USA 113, 7590–7595. 10.1073/pnas.1606303113 PMC4941467 27330113

[CIT0113] Ravenscroft CH, Fridley JD, Grime JP 2014 Intraspecific functional differentiation suggests local adaptation to long-term climate change in a calcareous grassland. Journal of Ecology 102, 65–73.

[CIT0114] Reed SC, Coe KK, Sparks JP, Housman DC, Zelikova TJ, Belnap J 2012 Changes to dryland rainfall result in rapid moss mortality and altered soil fertility. Nature Climate Change 2, 752–755.

[CIT0115] Reich PB 2014 The world-wide ‘fast–slow’ plant economics spectrum: a traits manifesto. Journal of Ecology 102, 275–301.

[CIT0116] Reymond M, Svistoonoff S, Loudet O, Nussaume L, Desnos T 2006 Identification of QTL controlling root growth response to phosphate starvation in *Arabidopsis thaliana*. Plant, Cell & Environment 29, 115–125. 10.1111/j.1365-3040.2005.01405.x 17086758

[CIT0117] Riboni M, Galbiati M, Tonelli C, Conti L 2013 GIGANTEA enables drought escape response via abscisic acid-dependent activation of the florigens and SUPPRESSOR OF OVEREXPRESSION OF CONSTANS. Plant Physiology 162, 1706–1719. 23719890 10.1104/pp.113.217729 PMC3707542

[CIT0118] Ruppert JC, Harmoney K, Henkin Z, Snyman HA, Sternberg M, Willms W, Linstädter A 2015 Quantifying drylands’ drought resistance and recovery: the importance of drought intensity, dominant life history and grazing regime. Global Change Biology 21, 1258–1270. 25407684 10.1111/gcb.12777

[CIT0119] Rus A, Baxter I, Muthukumar B, Gustin J, Lahner B, Yakubova E, Salt DE 2006 Natural variants of AtHKT1 enhance Na^+^ accumulation in two wild populations of *Arabidopsis*. PLoS Genetics 2, e210. 17140289 10.1371/journal.pgen.0020210 PMC1665649

[CIT0120] Sánchez-Bermejo E, Méndez-Vigo B, Picó FX, Martínez-Zapater JM, Alonso-Blanco C 2012 Novel natural alleles at FLC and LVR loci account for enhanced vernalization responses in *Arabidopsis thaliana*. Plant, Cell & Environment 35, 1672–1684. 10.1111/j.1365-3040.2012.02518.x 22494398

[CIT0121] Salt DE, Baxter I, Lahner B 2008 Ionomics and the study of the plant ionome. Annual Review of Plant Biology 59, 709–733. 10.1146/annurev.arplant.59.032607.092942 18251712

[CIT0122] Sasaki E, Zhang P, Atwell S, Meng D, Nordborg M 2015 ‘Missing’ G × E variation controls flowering time in *Arabidopsis thaliana*. PLoS Genetics 11, e1005597. 26473359 10.1371/journal.pgen.1005597 PMC4608753

[CIT0123] Savolainen O, Lascoux M, Merilä J 2013 Ecological genomics of local adaptation. Nature Reviews. Genetics 14, 807–820. 10.1038/nrg3522 24136507

[CIT0124] Segrestin J, Bernard-Verdier M, Violle C, Richarte J, Navas ML, Garnier E 2018 When is the best time to flower and disperse? A comparative analysis of plant reproductive phenology in the Mediterranean. Functional Ecology 7, 1770–1783.

[CIT0125] Shindo C, Aranzana MJ, Lister C, Baxter C, Nicholls C, Nordborg M, Dean C 2005 Role of FRIGIDA and FLOWERING LOCUS C in determining variation in flowering time of *Arabidopsis*. Plant Physiology 138, 1163–1173. 15908596 10.1104/pp.105.061309 PMC1150429

[CIT0126] Smith MD, Knapp AK, Collins SL 2009 A framework for assessing ecosystem dynamics in response to chronic resource alterations induced by global change. Ecology 90, 3279–3289. 20120798 10.1890/08-1815.1

[CIT0127] Stock AJ, McGoey BV, Stinchcombe JR 2015 Water availability as an agent of selection in introduced populations of *Arabidopsis thaliana*: impacts on flowering time evolution. PeerJ 3, e898. 25909038 10.7717/peerj.898 PMC4406364

[CIT0128] Svardal H, Farlow A, Exposito-Alonso M, Ding W, Novikova P, Alonso-Blanco C, Weigel D, Lee C-R, Nordborg M 2017 On the post-glacial spread of human commensal *Arabidopsis thaliana*. Nature Communications 8, 1–12. 10.1038/ncomms14458 PMC5309843 28181519

[CIT0129] Tabas-Madrid D, Méndez-Vigo B, Arteaga N, Marcer A, Pascual-Montano A, Weigel D, Picó FX, Alonso-Blanco C 2018 Genome-wide signatures of flowering adaptation to climate temperature: regional analyses in a highly diverse native range of *Arabidopsis thaliana*. Plant, Cell & Environment 41, 1806–1820. 10.1111/pce.13189 29520809

[CIT0130] Taylor MA, Cooper MD, Sellamuthu R, Braun P, Migneault A, Browning A, Perry E, Schmitt J 2017 Interacting effects of genetic variation for seed dormancy and flowering time on phenology, life history, and fitness of experimental *Arabidopsis thaliana* populations over multiple generations in the field. New Phytologist 216, 291–302. 28752957 10.1111/nph.14712

[CIT0131] Tomatsu H, Takano J, Takahashi H, Watanabe-Takahashi A, Shibagaki N, Fujiwara T 2007 An *Arabidopsis thaliana* high-affinity molybdate transporter required for efficient uptake of molybdate from soil. Proceedings of the National Academy of Sciences, USA 104, 18807–18812. 10.1073/pnas.0706373104 PMC2141858 18003916

[CIT0132] Toomajian C, Hu TT, Aranzana MJ, Lister C, Tang C, Zheng H, Zhao K, Calabrese P, Dean C, Nordborg M 2006 A nonparametric test reveals selection for rapid flowering in the *Arabidopsis* genome. PLoS Biology 4: e137. 16623598 10.1371/journal.pbio.0040137 PMC1440937

[CIT0133] van Heerwaarden J, van Zanten M, Kruijer W 2015 Genome-wide association analysis of adaptation using environmentally predicted traits. PLoS Genetics 11, e1005594. 26496492 10.1371/journal.pgen.1005594 PMC4619680

[CIT0134] Vasseur F, Exposito-Alonso M, Ayala-Garay OJ, Wang G, Enquist BJ, Vile D, Violle C, Weigel D 2018a. Adaptive diversification of growth allometry in the plant *Arabidopsis thaliana*. Proceedings of the National Academy of Sciences, USA 115, 3416–3421. 10.1073/pnas.1709141115 PMC5879651 29540570

[CIT0135] Vasseur F, Sartori K, Baron E, Fort F, Kazalou E, Segrestin J, Garnier E, Vile D, Violle C 2018b. Climate as a driver of adaptive variations in ecological strategies in *Arabidopsis thaliana*. Annals of Botany 122, 935–945. 30256896 10.1093/aob/mcy165 PMC6266113

[CIT0136] Vicca S, Gilgen AK, Camino Serrano M, et al 2012 Urgent need for a common metric to make precipitation manipulation experiments comparable. New Phytologist 195, 518–522. 22734795 10.1111/j.1469-8137.2012.04224.x

[CIT0137] Vidigal DS, Marques ACSS, Willems LAJ, Buijs G, Méndez-Vigo B, Hilhorst HWM, Bentsink L, Picó FX, Alonso-Blanco C 2016 Altitudinal and climatic associations of seed dormancy and flowering traits evidence adaptation of annual life cycle timing in *Arabidopsis thaliana*. Plant, Cell & Environment 39, 1737–1748. 10.1111/pce.12734 26991665

[CIT0138] Volaire F 2018 A unified framework of plant adaptive strategies to drought: crossing scales and disciplines. Global Change Biology 24, 2929–2938. 29350812 10.1111/gcb.14062

[CIT0139] Weigel D, Nordborg M 2015 Population genomics for understanding adaptation in wild plant species. Annual Review of Genetics 49, 315–338. 10.1146/annurev-genet-120213-092110 26436459

[CIT0140] Westoby M, Falster SD, Moles AT, Vesk PA, Wright IJ 2002 Plant ecological strategies: some leading dimensions of variation between species. Annual Review of Ecology, Evolution and Systematics 33, 125–159.

[CIT0141] Whittaker C, Dean C 2017 The FLC locus: a platform for discoveries in epigenetics and adaptation. Annual Review of Cell and Developmental Biology 33, 555–575. 10.1146/annurev-cellbio-100616-060546 28693387

[CIT0142] Wilczek AM, Roe JL, Knapp MC, et al 2009 Effects of genetic perturbation on seasonal life history plasticity. Science 323, 930–934. 19150810 10.1126/science.1165826

[CIT0143] Wuest SE, Niklaus PA 2018 A plant biodiversity effect resolved to a single locus. BioRxiv, 264960. [Preprint] 10.1038/s41559-018-0708-y PMC6252258 30397303

[CIT0144] Zou YP, Hou XH, Wu Q, et al 2017 Adaptation of *Arabidopsis thaliana* to the Yangtze River basin. Genome Biology 18, 239. 29284515 10.1186/s13059-017-1378-9 PMC5745794

